# NLRP3 inflammasome mediates contrast media-induced acute kidney injury by regulating cell apoptosis

**DOI:** 10.1038/srep34682

**Published:** 2016-10-10

**Authors:** Jianxiao Shen, Ling Wang, Na Jiang, Shan Mou, Minfang Zhang, Leyi Gu, Xinghua Shao, Qin Wang, Chaojun Qi, Shu Li, Wanpeng Wang, Xiajing Che, Zhaohui Ni

**Affiliations:** 1 Department of Nephrology, Renji Hospital, School of Medicine, Shanghai Jiaotong University, 1630 Dong Fang Road, Shanghai, 200127, China

## Abstract

Iodinated contrast media serves as a direct causative factor of acute kidney injury (AKI) and is involved in the progression of cellular dysfunction and apoptosis. Emerging evidence indicates that NLRP3 inflammasome triggers inflammation, apoptosis and tissue injury during AKI. Nevertheless, the underlying renoprotection mechanism of NLRP3 inflammasome against contrast-induced AKI (CI-AKI) was still uncertain. This study investigated the role of NLRP3 inflammasome in CI-AKI both *in vitro* and *in vivo*. In HK-2 cells and unilateral nephrectomy model, NLRP3 and NLRP3 inflammasome member ASC were significantly augmented with the treatment of contrast media. Moreover, genetic disruption of NLRP3 notably reversed contrast-induced expression of apoptosis related proteins and secretion of proinflammatory factors, similarly to the effects of ASC deletion. Consistent with above results, absence of NLRP3 in mice undergoing unilateral nephrectomy also protected against contrast media-induced renal cells phenotypic alteration and cell apoptosis via modulating expression level of apoptotic proteins. Collectively, we demonstrated that NLRP3 inflammasome mediated CI-AKI through modulating the apoptotic pathway, which provided a potential therapeutic target for the treatment of contrast media induced acute kidney injury.

Acute kidney injury (AKI) is a clinical syndrome with a quite high mortality and morbidity rate. With the widely use of iodinated contrast media in medical diagnosis and treatment of disease, the contrast-induced AKI (CI-AKI) has become the third common cause of hospital-acquired renal insufficiency, which ranks only after the decreased renal perfusion and nephrotoxic medications^1^. The morbidity of CI-AKI in patients with normal renal function is 5%, while which is up to 20% among the patients with high risk (including aged, renal insufficiency, diabetes mellitus, cardiac insufficiency, combination of renal toxicity of drugs, *et al*.)^2^. KDIGO clinical practice guideline for acute kidney injury indicates that CI-AKI might lead to the prolongation of hospitalization, higher rate of cardiovascular events and increased all-cause mortality^3^. Although increasing studies focused on the physiological and pathological processes of CI-AKI, the pathogenesis of CI-AKI is still unclear.

Increasing evidences show that inflammatory response is involved in apoptosis and cellular dysfunction during AKI, mediated by the activation of inflammasome. These protein complexes act as a vital component in innate immunity through activating the proinflammatory cytokines interleukin (IL)-1β and IL-18^4^,^5^,^6^. Among multi-type of identified inflammasomes, the Nucleotide-binding oligomerization domain [NOD]-like pyrin domain containing protein 3 (NLRP3) inflammasome is the best characterized and can be activated by diverse stimuli including pathogen-associated molecular patterns (PAMPs) and danger-associated molecular patterns (DAMPs)^7^,^8^,^9^. Activated NLRP3 could oligomerize and recruit apoptosis-associated speck-like protein containing a caspase recruitment domain [CARD] (ASC) as well as caspase-1, formatting NLRP3 inflammasome. Caspase-1, with inflammation-promoting effect, is activated by NLRP3 inflammasome and further dissociated proIL-1β and proIL-18 into the mature and active form, which further then induced inflammatory response^9^.

The activation of NLRP3 inflammasome has been shown to contribute to the inflammatory response of various models of AKI, such as ischemic-induced AKI, rhabdomyolysis-induced AKI, and sepsis-induced AKI^10^,^11^,^12^. Whether NLRP3 inflammasome participates in the pathogenetic process of CI-AKI still remains unclear. Certain studies have demonstrated that the enhanced number of apoptotic cells is a crucial and characteristic feature of AKI^13^,^14^. In the current study, we proposed to investigate the effect of NLRP3 inflammasome activation on CI-AKI pathogenesis and the underlying mechanism through evaluating proximal renal tubular epithelial cell apoptosis *in vitro* and *in vivo*.

## Results

### Contrast media increases the expression levels of NLRP3 and ASC in HK-2 cell

**Figure 1:**
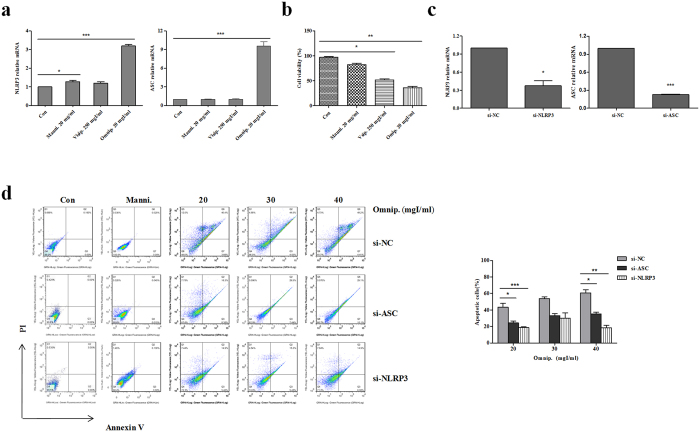
si-NLRP3 or si-ASC effect on omnipaque-induced cell apoptosis in HK-2 cells. **(a)** Quantitative RT-PCR of NLRP3 and ASC. Cells were treated with 20 mg/ml mannitol (Manni.), 250 mgI/ml visipaque (Visip.), and 20 mgI/ml omnipaque (Omnip.) respectively for 72 h. **(b)** Quantification of cell viability by flow cytometry. HK-2 cells were treated with 20 mg/ml mannitol (Manni.), 250 mgI/ml visipaque (Visip.), and 20 mgI/ml omnipaque (Omnip.) respectively for 72 h. **(c)** Quantitative RT-PCR analysis of NLRP3 expression in si-NLRP3 cell and ASC expression in si-ASC cell, with si-NC (non-specific control) cell as control. **(d)** Quantification of cell apoptosis by flow cytometry. HK-2 cells transfected with si-NC or si-NLRP3 or si-ASC were treated with mannitol (Manni., 20 mg/ml), omnipaque (Omnip., 20–40 mgI/ml). si-ASC and si-NLRP3 dramatically attenuated omnipaque-induced cell apoptosis. Bars represent percentages of apoptotic cells according to the left data. Data are shown as mean ± SD. *P < 0.05, **P < 0.01, ***P < 0.001.

To evaluate the role of NLRP3 and ASC in CI-AKI, we first determined whether the levels of NLRP3 and ASC mRNA were influenced by contrast media in HK-2 cell. By applying quantitative RT-PCR, we examined the mRNA expression levels of NLRP3 and ASC in HK-2 cells treated by 20 mg/ml mannitol, 250 mgI/ml visipaque. 20 mgI/ml omnipaque. respectively for 72 h. Obviously, NLRP3 and ASC were remarkably induced by omnipaque (P < 0.001) compared to the other two kinds of control treatments (Fig. 1a). Meanwhile, the level of NLRP3 mRNA partly increased with 20 mgI/ml mannitol treatment for 72 h, whereas, ASC mRNA nearly remained at the same level with or without mannitol treatment. Further, the results of flow cytometry indicated that cell viability was obviously decreased under the treatments of visipaque (P < 0.05) and omnipaque (P < 0.01) (Fig. 1b). Thus, omnipaque, with mannitol as a control, was chosen as the contrast media which used for stimulating AKI for our future *in vitro* experiments.

### Silence of NLRP3 or ASC attenuates contrast-induced apoptosis in HK-2 cell

Given that iodinated contrast media induced AKI, we first investigated whether contrast media can induce injury in HK-2 cell. Firstly, we silenced NLRP3 and ASC via a siRNA strategy, and quantitative RT-PCR results showed that NLRP3 and ASC were deleted successfully in HK-2 cell (Fig. 1c). Afterwards, we determined omnipaque effects on si-NLRP3 HK-2 cells and si-ASC HK-2 cells using annexin V/flow cytometry. As shown in Fig. 1d, omnipaque treatment induced cell apoptosis in a dose-dependent manner compared with mannitol treatment. With the increased dose of omnipaque, the number of apoptotic cells in si-NLRP3 and si-ASC were both more than mannitol treated cells. Moreover, with 20 mgI/ml and 40 mgI/ml omnipaque treatment, si-NLRP3 dramatically ameliorated omnipaque-induced cell apoptosis compared with si-NC (P < 0.01 and P < 0.001, respectively). Whereas, si-ASC decreased omnipaque-induced cell apoptosis a little (P < 0.05).

**Figure 2:**
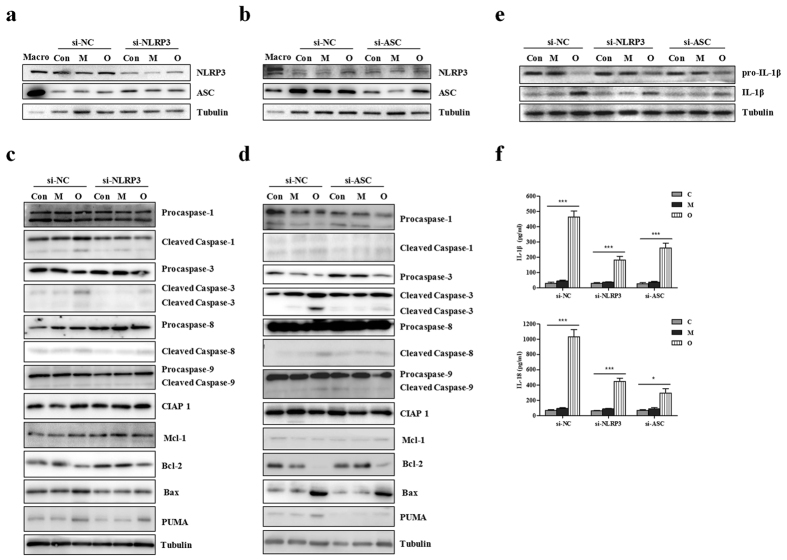
si-NLRP3 or si-ASC effect on the expressions of apoptosis associated proteins and levels of IL-1β and IL-18 in HK-2 cells responded to different kinds of contrast media. (**a**) Western blot of NLRP3 and ASC in si-NC (non-specific control) and si-NLRP3. Macrophages are shown as positive controls (Macro). (**b**) Western blot of NLRP3 and ASC in si-NC (non-specific control) and si-ASC. Macrophages are shown as positive controls (Macro). (**c**) Western blot of procaspase-1,−3,−8,−9, cleaved caspase-1,−3,−8,−9, other apoptotic proteins (CIAP 1, Mcl-1, Bcl-2, Bax and PUMA) in si-NC (non-specific control) and si-NLRP3. (**d**) Western blot of procaspase-1,−3,−8,−9, cleaved caspase-1,−3,−8,−9, other apoptotic proteins (CIAP 1, Mcl-1, Bcl-2, Bax and PUMA) in si-NC and si-ASC. (**e**) Western blot of pro-IL-1β and IL-1β in si-NC, si-NLRP3 and si-ASC. (**f**) ELISA assay of the levels of IL-1β and IL-18. HK-2 cells transfected with si-NC or si-NLRP3 or si-ASC were treated with mannitol and omnipaque, respectively. Data are shown as mean ± SD. *P < 0.05, ***P < 0.001.

Next, a correlation analysis with western blotting was conducted to detect whether silence of NLRP3 or ASC would modulate activation of their downstream component caspase-1 and accumulation of apoptosis related proteins. As shown in Fig. 2a,b, either si-NLRP3 or si-ASC could significantly inhibit omnipaque-induced increase of NLRP3 protein level. Strikingly, omnipaque-induced up-regulation of cleaved caspase-1 was decreased in si-NLRP3 and si-ASC (Fig. 2c,d). Furthermore, omnipaque-induced suppression of anti-apoptotic proteins (CIAP1 and Bcl-2) was inhibited by NLRP3 or ASC gene knockdown (Fig. 2c,d). Meanwhile, omnipaque-induced accumulation of pro-apoptotic proteins (PUMA and Bax), activation of death initiator caspase-8, as well as activation of executioner caspase-3 were reduced by NLRP3 or ASC gene deletion (Fig. 2c,d). However, activation level of caspase-9 had no obvious alteration in si-NLRP3 cells and si-ASC cells, compared to si-NC cells. These results suggest that NLRP3 inflammasome may be a pathogenic factor mediating iodinated contrast media induced apoptosis *in vitro*.

### Silence of NLRP3 or ASC alleviates contrast-induced secretion of IL-1β and IL-18 in HK-2 cell

As shown above, the inhibition of NLRP3 or NLRP3 inflammasome member ASC down-regulated the level of inflammatory factors. Currently, we measured the secretion of another two indicators of inflammasome activation, namely the proinflammatory cytokines IL-1β and IL-18. Using Western blotting, we found that silencing of NLRP3 or ASC could modulated contrast media induced IL-1β processing (Fig. 2e). With Elisa assay, we found that contrast media-induced secretion of IL-1β and IL-18 were both severely alleviated by the deletion of NLRP3 or ASC (Fig. 2f). Taken together, NLRP3 inflammasome plays a vital role in mediating contrast-induced apoptosis and inflammatory response *in vitro*.

### Contrast media increases the expression levels of NLRP3 and ASC *in vivo*

**Figure 3:**
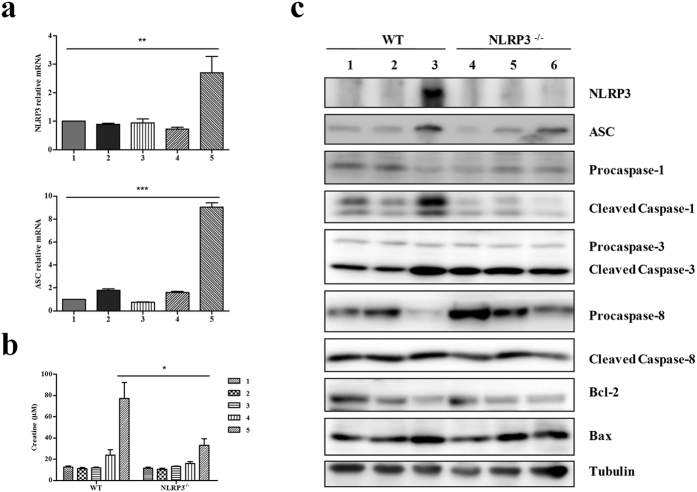
NLRP3 deficiency effect on the expressions of apoptosis associated proteins *in vivo* responded to contrast media. (**a**) qRT-PCR of NLRP3 and ASC in NLRP^−/−^ mice following different treatments (n = 4 per group). 1. control; 2. model undergoing unilateral nephrectomy; 3. model + water deprivation for one day; 4. model + water deprivation for one day + furosemide injection; 5. model + water deprivation for one day + furosemide injection + contrast media. **(b)** Analysis of serum creatinine level in wild type (WT) mice and NLRP3^−/−^ mice following different treatments (n = 4 per group). 1. control; 2. model undergoing unilateral nephrectomy; 3. model + water deprivation for one day; 4. model + water deprivation for one day + furosemide injection; 5. model + water deprivation for one day + furosemide injection + contrast media. **(c)** Western blot of NLRP3, ASC, procaspase-1,−3, −8 s, cleaved caspase-1,−3,−8 s, Bcl-2, and Bax in NLRP3^−/−^ mice (n = 4 per group). 1, 4. control; 2, 5. model + water deprivation for one day + furosemide injection; 3, 6. model + water deprivation for one day + furosemide injection + contrast media. Data are shown as mean ± SD. *P < 0.05, ***P < 0.001.

Subsequently, we examined whether activation of NLRP3 inflammasome could modulate CI-AKI *in vivo*. Quantitative RT-PCR was conducted to supervise alteration of NLRP3 and ASC mRNA level responding to contrast media in mice undergoing unilateral nephrectomy. Compared with the control groups, both NLRP3 expression and ASC expression were significantly increased with contrast media treatment (Fig. 3a). These data indicate that contrast media regulated expression levels of NLRP3 and ASC *in vivo*.

### NLRP3 deficiency modulates CI-AKI and apoptosis *in vivo*

To confirm contrast media resulted in AKI, we firstly detected the serum creatinine level in mice undergoing unilateral nephrectomy following different treatments, including water deprivation for one day, furosemide injection, contrast media. Obviously, the level of serum creatinine in wild type (WT) mice treated with water deprivation, furosemide injection and contrast media was dramatically higher than other groups, and this effect of contrast media was partly attenuated by the deficiency of NLRP3 in mice (Fig. 3b).

Considering the changed level of NLRP3 inflammasome downstream apoptosis related proteins in si-NLRP3 HK-2 cells, we further verified these *in vivo*. Consistent with the results in HK-2 cells, contrast media-induced cleavages of caspase-1,−3,−8 were obviously attenuated in NLRP3^−/−^ mice undergoing unilateral nephrectomy. Accordingly, accumulation of pro-apoptotic protein Bax promoted by contrast media was inhibited by deletion of NLRP3. On the contrary, suppressed expression of anti-apoptosis protein Bcl-2 was ameliorated by NLRP3 deficiency under contrast media treatment. Meanwhile, the expression of NLRP3 inflammasome member ASC decreased as well (Fig. 3c). These results suggest that NLRP3 inflammasome mediates CI-AKI and apoptosis.

### NLRP3 inflammasome affects contrast-induced renal injury *in vivo*

**Figure 4:**
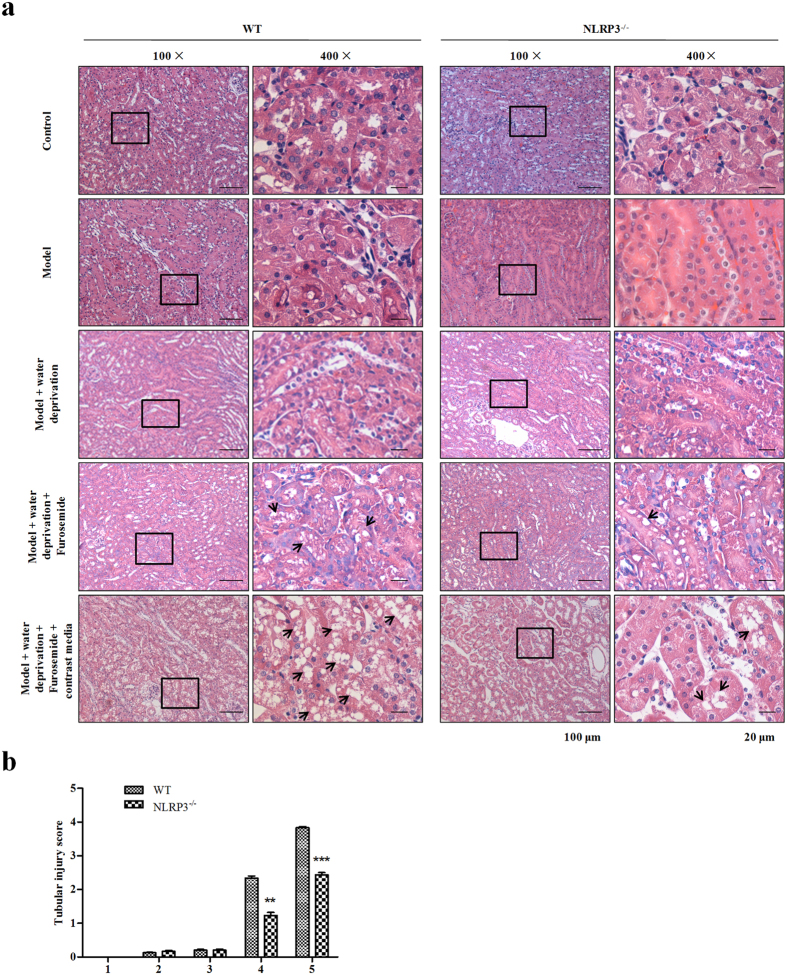
NLRP3 deficiency effect on contrast-induced histological injury *in vivo*. (**a**) Represented image of hematoxylin and eosin (H&E) staining in the tubular (black arrows indicating the injury). (**b**) Tubular injury score in the outer medulla was determined. 1. control; 2. Model undergoing unilateral nephrectomy; 3. Model + water deprivation for one day; 4. Model + water deprivation for one day + Furosemide; 5. Model + water deprivation + Furosemide + contrast media. Data are shown as mean ± SD. **P < 0.01, ***P < 0.001.

**Figure 5:**
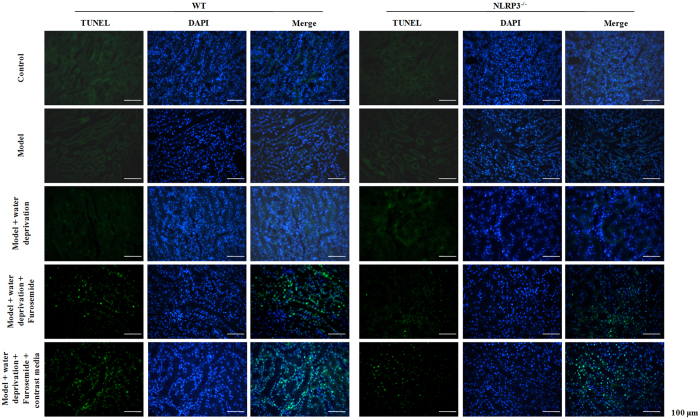
NLRP3 deficiency effect on contrast-induced cell apoptosis *in vivo*. *In situ* cell death detected by TUNEL assay and DAPI staining.

To explore the effect of NLRP3 on CI-AKI, Hematoxylin-eosin (H&E) staining was conducted. We found that contrast media induced remarkable renal structure damage, such as inflammatory cellular infiltration, extensive tubular vacuolization, tubular epithelial cell exfoliation and thickening of glomerular basement membrane in WT mice and these alterations were notably alleviated in NLRP3^−/−^ mice, which indicated that renal injury was significantly hampered in NLRP3^−/−^ mice compared with the controls (Fig. 4). With TUNEL staining, we found that contrast media dramatically aggravated apoptosis in the kidney of mice underdoing unilateral nephrectomy. Furthermore, the effect of contrast media was prominently ameliorated by the deletion of NLRP3 gene in mice (Fig. 5).

**Figure 6:**
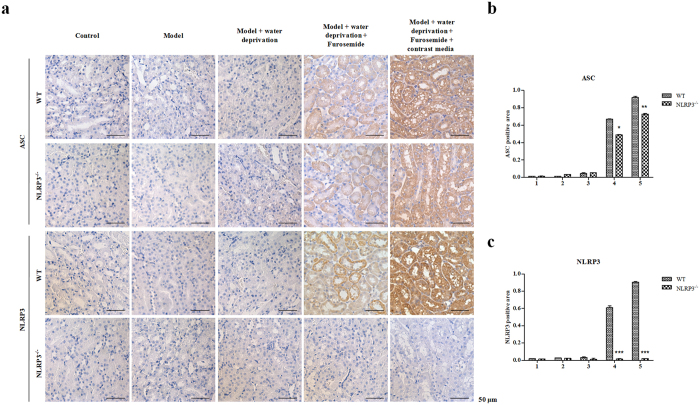
NLRP3 deficiency effect on contrast-induced expressions of NLRP3 and ASC *in vivo*. (**a**) NLRP3 deficiency effect on contrast-induced expression of ASC and NLRP3 *in vivo* detected by Immunohistochemistry (IHC). (**b**) A quantitative analysis of ASC positive area in the cortex and outer medulla. (**c**) A quantitative analysis of NLRP3 positive area in the cortex and outer medulla. Data are shown as mean ± SD.

Using IHC staining, we monitored the levels of NLRP3 and inflammasome components ASC (Fig. 6). The amount of NLRP3 was comparable between contrast media treated NLRP3^−/−^ mice and WT mice. In the WT mice undergoing unilateral nephrectomy, contrast media significantly promoted the expression of NLRP3. As expected, the expression of NLRP3 was deleted by deficiency of NLRP3 (Fig. 6c). Moreover, ASC level was significantly decreased in NLRP3^−/−^ mice compared to the WT mice with contrast media treatment, suggesting that the renal damage was due to the activation of NLRP3 inflammasome (Fig. 6b). These data suggest that NLRP3 inflammasome medias CI-AKI through modulating apoptosis.

## Discussion

Contrast media, might giving rise to renal hypoxic-ischemic, oxidative stress or direct cytotoxicity of itself and so on, has been supposed to be a causative and aggravating factor of progressive AKI^15^,^16^,^17^. Renal tubular injury caused by renal hypoxic-ischemic was regarded as the main pathogenesis of CI-AKI. After the administration of contrast media, blood viscosity were forced to rise and resulted in the increased blood flow resistance and reduced renal blood flow. And the higher blood viscosity also contributed to the elevation of initial urine viscosity, obstruction of renal tubules, and further augmented the interstitial pressure and aggravated medulla hypoperfusion^15^. The viscosity of contrast medium had nothing to do with its osmotic pressure, and compared with hypotonic contrast media, the isotonic contrast media exerts more significant effect on renal medulla oxygen partial pressure during the mice experiments^18^. However, the orgin of cytotoxicity of contrast media is still unknown. As observed *in vitro* cytotoxicity-test, the cytotoxicity of different types of contrast media such as non-ionic hypotonic contrast media, non-ionic isotonic contrast media and ionic contrast media for renal tubular epithelial cell was increased in sequence^19^. Here, we determined NLRP3 and ASC expression levels in HK-2 cells with diverse types of contrast media and found that non-ionic hypotonic contrast media exerts more significant modulating effects on NLRP3 and ASC (Fig. 1a).

As mentioned earlier, oxidative stress was another hypothetical pathogenesis of CI-AKI. Oxygen deficit in medulla kidney could accelerate the generation of local reactive oxygen species (ROS). Contrast media-induced debasement of glomerular filtration rate could be suppressed by inhibiting or decreasing the generation of ROS, which was found in animal studies^20^. Tepel *et al*. indicated that prophylactic oral administration of the antioxidant acetylcysteine would effectively diminish the morbidity of CI-AKI in patients with chronic kidney insufficiency^21^, whereas it has not been verified by any subsequent clinical trial. It has been reported that all kinds of contrast media reduce the proliferation of renal tubular epithelial cells cultured *in vitro*, which suggested that the effect of cytotoxicity was possessed by contrast media^19^. Contrast media may destroy mitochondrial enzyme activity and mitochondrial membrane potential of renal tubular epithelial cell, and then result in the release of cytochrome c and initiating of cell apoptosis process^19^. Moreover, hemeoxygenase-1 could ameliorate AKI through modulating the activity of apoptosis associated proteins, including Bcl-2, Bax, caspase-3 and caspase-9^22^. Reduced level of anti-apoptotic proteins, such as Bcl-2, and increased level of pro-apoptotic proteins, such as Bax were detected in cis-induced AKI^23^. In the present study, we found that treatment with contrast media resulted in the down-regulation or degradation of anti-apoptotic proteins, such as Bcl-2 and caspase-3, whereas the expression level of pro-apoptotic protein Bax was ascendant. Importantly, all these results were reversed in the deficiency of NLRP3 both *in vitro* and *in vivo*.

To date, the detrimental role of NLRP3 in some kidney injury models have been reported. NLRP3 inflammasome in renal tissues could be activated by unilateral ureteral obstruction. Compared with wild type mice, NLRP3 gene knockout mice got alleviated kidney tubules damage and inflammatory response^24^. Ischemia reperfusion (IR) induced the expression of NLRP3 in mice renal tissues, and NLRP3 gene knockout mouse model can remarkably ameliorate IR-induced AKI including acute tubule necrosis, expression of IL-18 and IL-1β, alleviate impaired function and reduce mortality^8^,^25^. It has been reported that lipopolysaccharide, as an activator of NLRP3, improved the NLRP3-caspase-1 axis activity in kidney dendritic cell cytoplasm and stimulate the secretion of IL-1β^26^. Previous study on hyperhomocysteinemia kidney damage revealed that NLRP3, ASC, caspase-1 all expressed in mice sertoli cell, and homocysteine activated the NLRP3 inflammasome, followed by cytoskeleton rearrangement^27^. In the present study, HK-2 cells with NLRP3 or ASC silence both obtained impaired NLRP3 inflammasome activation and suppressed pro-apoptotic protein, as well as increased anti-apoptotic proteins. Whether the effect of NLRP3 on CI-AKI requires ASC or not is unclear. Furthermore, NLRP3 deletion studies in mice undergoing unilateral nephrectomy suggested that NLRP3 plays a vital role on contributing to contrast media-induced acute kidney injury and cell apoptosis as confirmed by both *in vitro* and *in vivo* studies.

Our *in vitro* cells-based study showed that NLRP3 inflammasome was associated with cell apoptosis in CI-AKI (Fig. 1c). This was consistent with the fact that the disruption of NLRP3 inflammasome was correlated with the expression and activation of apoptosis associated proteins (Fig. 2a,b). *In vivo*, our data presented the decisive role of NLRP3 inflammasome in contrast-induced cell morphology and cell mortality (Figs 4 and 5). Caspase-1,−3,−8, Bcl-2 and Bax were all involved in the anti-apoptotic effects of NLRP3 inflammasome in contrast media-induced renal injury (Fig. 3c).

The current study still exits several limitations. First, NLRP3 exerted an inflammasome independent effect on ameliorating TGR-β1-induced epithelial-to-mesenchymal transition (EMT) of proximal renal tubular epithelial cell^28^. We also determined the level of the EMT markers Collagen III and E-cad in si-NLRP3 and si-ASC with omnipaque treatment. However, these candidate markers might involved in CI-AKI did not respond to NLRP3 or ASC silence (data not shown). Second, to detect the effect of NLRP3 inflammasome on CI-AKI, we constructed two kinds of HK-2 cells, namely si-NLRP3 cell and si-ASC cell. Whereas, we only obtained NLRP3^−/−^ mice and WT mice for investigating apoptosis and cell phenotype change during CI-AKI. We could not speculate whether the NLRP3 reversed contrast media induced effects in a NLRP3 inflammasome dependent way or not. Third, although the NLRP3 expression was inhibited via gene knockout technique in *in vivo* studies, we could not rule out the limitations of the genetic inhibition. Thus, further studies will necessary to verify the role of NLRP3 in the pathogenesis of CI-AKI and highlight its clinical value through adding NLRP3 pharmacological inhibitor, for example, Arglabin^29^, to the *in vivo* researches.

In conclusion, we herein reported that the NLRP3 inflammasome exerted primary effects on CI-AKI by modulating the cell apoptosis, thus provided a novel therapeutic target for kidney diseases, particularly for the treatment of CI-AKI.

## Methods

### Cell culture and mice preparation

si-RNA transfection Cell line HK-2 (human renal proximal tubular) was obtained from the American Type Culture Collection (ATCC, MA, USA). And cells were cultured at 37 °C with a humidified atmosphere of 95% O_2_ and 5% CO_2_ using Dulbecco’s Modified Eagle’s medium containing 10% fatal bovine serum (FBS) (Invitrogen, Carlsbad, CA, USA), 100 U/ml penicillin, 100 μg/ml streptomycin and various agents including 20 mg/ml Mannitol (hypertonic control solution, 1098 mOsm/kg H_2_O, EFEBIO, Shanghai, China), 250 mgI/ml Visipaque (non-ionic isotonic contrast media, 290 mOsm/kg H_2_O, GE Healthcare, Shanghai, China), or 20–40 mgI/ml Omnipaque (non-ionic hypotonic contrast media, 844 mOsm/kg H_2_O, GE Healthcare, Shanghai, China), respectively for 72 h.

For gene knockdown studies, NLRP3 siRNA, ASC siRNA, and nonspecific (NC) siRNA were synthesized by Bioeverest Technology (Shenzhen, China). HK-2 cells were then transfected with si-NLRP3 or si-ASC or si-NC 24 h before contrast media treatment. And iodinated contrast media was used to stimulate HK-2 cells by reference to previous study^30^. NLRP3^−/−^ and wild type male mice on C57BL/6 background (Jackson Lab) were housed under standard conditions with a 12-h light/12-h dark cycle at 22–24 °C and allowed free access to water and food. All mice were randomized into four groups: (1) Group A, control (n = 6); (2) Group B, model undergoing unilateral nephrectomy (n = 6); (3) Group C, model + water deprivation for one day (n = 6); (4) Group D, model + water deprivation for one day + furosemide injection (10 μl/g) (n = 6); (5) Group E, model + water deprivation for one day + furosemide injection (10 μl/g) + contrast media (10 μl/g Omnipaque, 350 mgI/ml, 844 mOsm/kg H_2_O) (n = 6). Furosemide and contrast media were injected via tail vein administration over each course of 5 min. The eyeballs of mice were removed 24 h after the last contrast media injection, and then their blood and renal tissues were observed.

### Ethics statement

All procedures involving these animals were approved by the Animal Protocol Committee of Shanghai Jiaotong University and conducted according to the Animal Care Committee at the Renji Hospital, School of Medicine, Shanghai Jiaotong University.

### Quantitative real time PCR

Total RNA was collected using TRI Reagent RNA Isolation Reagent (Sigma-Aldrich, St. Louis, MO, USA). First strand cDNA was synthesized with AMV reverse transcriptase (Amresco, Solon, HO, USA). Quantitative RT-PCR was performed in the 7500 Fast Real Time PCR System (Applied Biosystems, Rockford, IL, USA) with SYBR Green PCR Master Mix (Roche, Netley, NJ, USA).The primer sequences for quantitative RT-PCR were as follows: NLRP3-F, (5′ACATCTCCTTGGTCCTCAGC 3′) and NLRP3-R, (5′GCTTCAGTCCCACACACAGA 3′); ASC-F, (5′CTGACGGATGAGCAGTACCA 3′) and ASC-R, (5′AAGTCCTTGCAGGTCCAGTT 3′). The following PCR condition was applied: 95 °C for 10 min, 40 cycles at 95 °C for 15 sec, and 60 °C for 1 min. Relative gene expression was normalized to 18 S rRNA as the internal control and calculated using the comparative CT method.

### Annexin V-Fluorescein Isothiocyanate Conjugated Propidium Iodide Staining

For characterizing the phenotype of HK-2 cells, cells were incubated with annexin V –fluorescein isothiocyanate (FITC) and propidium iodide (PI) according to the manufacturer’s instruction of annexinV:FITC apoptosis detection kit (Sigma, St. Louis, MO, USA). Quantification was then conducted by a FACSCalibur flow cytometer using CellQuest software (BD Biosciences, San Jose, CA, USA).

### Western blotting

HK-2 cells and mice tissues were lysed using a protein lysis buffer containing 20 mM Tris (pH 7.4), 150 mM NaCl, 1 mM EDTA, 1 mM EGTA, 1% Triton X-100, 25 mM sodium pyrophosphate, and 2 mM sodium orthovanadate aprotinin. All denatured proteins were separated by SDS-PAGE gel and transferred to polyvinylidene difluoride membranes (Roche, Netley, NJ, USA). The membranes were blocked with 5% skimmed milk in Tris-buffered saline and then incubated with 1: 500 dilutions of primary antibodies as follows: anti-NLRP3 (Proteintech, Chicago, IL,USA), anti-ASC (Proteintech, Chicago, IL,USA), anti-Caspase-1 (Abcam, Cambridge, MA, US), anti-Cleaved Caspase-1 (Santa, Santa Cruz, CA, USA), anti-Caspase-3 (CST, Beverly, MA, USA), anti-Cleaved Caspase-3 (CST, Beverly, MA, USA), anti-Caspase-8 (CST, Beverly, MA, USA), anti-Cleaved Caspase-8 (CST, Beverly, MA, USA), anti-Caspase-9 (CST, Beverly, MA, USA), anti-Cleaved Caspase-9 (CST, Beverly, MA, USA), anti-Collagen III (Abcam, Cambridge, MA, US), anti-E-card (Abcam, Cambridge, MA, US), anti-CIAP 1 (Abcam, Cambridge, MA, US), anti-Mcl-1 (Abcam, Cambridge, MA, US), anti-Bcl-2 (Antibody Revolution, San Diego, CA, USA), anti-Bax (Abcam, Cambridge, MA, US), anti-PUMA (Abcam, Cambridge, MA, US), and anti-Tubulin (Proteintech, Chicago, IL,USA). The samples were then incubated with horseradish peroxidase-conjugated anti-rabbit secondary antibody (R&D Systems China Co. Ltd, Shanghai, China).The bands were visualized using the ECL Western Blotting Kit (Biovision, Milpitas, CA, USA) and quantified by Quantity One software (Bio-Rad, Hercules, CA, USA).

### ELISA

The levels of IL-1β and IL-18 were detected with TMB enzyme-linked immunosorbent assay (ELISA) kit (Invitrogen, GIBCO, Carlsbad, CA, USA) according to the manufacturer’s instructions. The absorbance was measured by a microplate reader (Bio-Rad, Hercules, CA, USA).

### Serum creatinine analysis

All serum samples were collected from mice in different groups and further analyzed by a standard spectrophotometric assay (Roche Diagnostics GmbH, Mannheim, Germany).

### Histopathology analysis

The renal tissue harvested from animal was washed with 0.9% saline, fixed in 10% netural buffered formalin and then embedded in 10% paraffin. Sections (5 μm thick) were stained with hematoxylin-eosin (H&E) or Masson trichrome reagents (Ambion, Carlsbad, CA, USA) for further microscopic analysis. The tubular injury score was calculated according to the following grades: grade 0, normal; grade 1, <25%; grade 2, 25–49%; grade 3, 50–74%; grade 4, ≥75%.

### *In situ* apoptosis assay

Terminal deoxynucleotidyl transferase-mediated deoxyuridine triphosphate nick end labeling (TUNEL) assay was performed to detect cell apoptosis in cultured cells or renal tissues using an *in situ* cell death detection kit (Roche, Netley, NJ, USA) according to the manufacturer’s protocol. Brown labeled TUNEL positive cells were counted in ten high-power (×400) fields.

### Immunohistochemistry

Paraffin-embedded sections were employed to deparaffinize and rehydrate the tissues. Then sections were microwaved in 0.01 M citrate buffer and washed with PBS for 15 min, and then treated with blocking buffer containing 5% BSA for 20 min at room temperature. Afterwards, the sections were incubated for 1 h at 37 °C with rabbit polyclonal antibodies anti-NLRP3 (1:1000) (Proteintech, Chicago, IL,USA), anti-ASC (1:500) (Proteintech, Chicago, IL,USA), and anti-caspase-3 (1:500) (Abcam, Cambridge, MA, US). After washing with PBS for 3 times, the secondary antibody was added, and immunostaining was performed using a DAB kit (Invitrogen, GIBCO, Carlsbad, CA, USA), and counterstain hematoxylin (Amresco, Solon, HO, USA). The ASC positive area and NLRP3 positive area were measured using ImageJ (U.S. National Institute of Health, Bethesda, MD).

### Statistical analysis

Data were expressed as mean ± standard deviation (SD). An unpaired *t* test was used to compare two groups. ANOVA with Tukey’s post test was used to assess the statistical significance between-group means for comparisons between multiple groups. The differences were considered statistically significant when P value < 0.05.

## Additional Information

**How to cite this article**: Shen, J. *et al*. NLRP3 inflammasome mediates contrast media-induced acute kidney injury by regulating cell apoptosis. *Sci. Rep.* **6**, 34682; doi: 10.1038/srep34682 (2016).
